# Some New and Unexpected Tauopathies in Movement Disorders

**DOI:** 10.1002/mdc3.12995

**Published:** 2020-08-03

**Authors:** Eoin Mulroy, Zane Jaunmuktane, Bettina Balint, Roberto Erro, Anna Latorre, Kailash P. Bhatia

**Affiliations:** ^1^ Department of Clinical and Movement Neurosciences UCL Queen Square Institute of Neurology London United Kingdom; ^2^ Division of Neuropathology, The National Hospital for Neurology and Neurosurgery University College London Hospitals National Health Service Foundation Trust London United Kingdom; ^3^ Department of Neurology University Hospital Heidelberg Germany; ^4^ Department of Medicine, Surgery and Dentistry "Scuola Medica Salernitana," Neuroscience Section University of Salerno Baronissi Italy

**Keywords:** neuropathology, tauopathies, movement disorders

Tauopathies are a heterogeneous group of neurodegenerative disorders sharing the neuropathologic hallmark of neuronal and/or glial accumulation of tau (Table 1). They can have genetic, toxic, autoimmune, or environmental bases, although in most cases, the etiology remains unknown. Recent years have seen increasing numbers of movement disorders subsumed under the umbrella of “tauopathies.” Herein we review these entities from clinical, etiological, and pathomechanistic standpoints and conclude by discussing the difficulties in interpreting the relevance of tau deposition in these disorders.

**TABLE 1:** *The known tauopathies—clinical presentation, causative mechanisms, and neuropathologic findings*

Disorder	Clinical Summary	Tau Isoforms	Tau Neuropathology	Usual Causative Mechanisms
**ADCY5‐related dyskinesia** ^a^	Infantile to adolescent onset choreo‐dystonia and/or myoclonus affecting the face, limbs, and/or neck. May be associated with axial hypotonia, facial twitching, and nocturnal exacerbations‐“ballistic bouts”	Mixed 3R and 4R	**Appearance:** NFTs, neuritic tau, and glial tau deposition **Anatomic Distribution:** Cerebral cortex, midbrain, thalamus and hippocampus. Sulcal perivascular astroglial tau inclusions also seen, though not typical of CTE	Genetic: *ADCY5* gene mutation
**Aging‐related tau astrogliopathy (ARTAG)**	ARTAG describes a spectrum of astroglial tau pathologies which are mainly encountered in the elderly, may occur in isolation or associated with other tauopathies and whose clinical relevance when an isolated finding is uncertain	4R‐tau	**Appearance**: Hyperphosphorylated tau accumulation in astrocytes, mainly taking the form of thorn‐shaped astrocytes and/or granular/fuzzy astrocytes **Anatomic Distribution:** Various spatial distributions of ARTAG have been defined, including subependymal, subpial, WM and GM ARTAG. These involve lobar, subcortical and brainstem regions with variable frequency depending on the subtype	Uncertain
**Alzheimer's disease (AD)**	Late‐onset AD: Progressive cognitive dysfunction characterized by memory loss, executive dysfunction, language dysfunction and impaired visuospatial ability Early‐onset (<65 years) disease can have atypical presentations with less memory impairment and more focal cortical syndromes	Mixed 3R and 4R	**Appearance:** NFTs (paired helical filaments and straight filaments), accompanied by widespread amyloid‐beta pathology, and hippocampal granulovacuolar degeneration and Hirano bodies **Anatomic Distribution:** Predictable spread of tau pathology over time from the transentorhinal cortex to the temporal allocortex, temporal neocortex and later to additional neocortical areas	Most commonly idiopathic neurodegeneration. Some familial syndromes due to *APP* and presenilin gene mutations
**Amyotrophic lateral sclerosis (ALS) and parkinsonism–dementia complex of Guam**	Atypical parkinsonism, dementia, motor neuron disease, or a combination of these 3 phenotypes, occurring both in the indigenous Chamorro population and in immigrants to the Mariana islands, the Kii peninsula of Japan, and the coastal plain of West New Guinea	Mixed 3R and 4R	**Appearance:** Dystrophic neurites, neurofibrillary tangles, coiled bodies and astrocytic inclusions. A lesser degree of Lewy body pathology affecting the SNR, LC and amygdala **Anatomic Distribution:** Neuronal and glial tau pathology in the neocortex, hippocampus, brainstem and spinal cord	Uncertain: possibly mediated through ingestion of environmental toxin
**Anti‐IGLON5 disease**	Sleep changes (OSA, parasomnia, goal‐directed movements during sleep), cognitive decline, movement disorders (chorea, parkinsonism, PSP‐like syndrome)	Mixed 3R and 4R	**Appearance:** Selective neuronal involvement with NFT, pretangles and neuropil threads **Anatomic Distribution:** Hypothalamus and brainstem tegmentum following rostro‐caudal severity gradient all the way down to the level of the cervical spinal cord	Autoimmune‐Anti‐IGLON5 antibodies
**Argyrophilic grain disease (AGD)**	Late‐onset slowly progressive MCI, sometimes with neuropsychiatric symptoms	4R‐tau	**Appearance:** In addition to argyrophilic grains and bush‐like astrocytes, tau inclusions consisting of oligodendrocytic coiled bodies (also seen in other 4R tauopathies such as PSP and CBD) and neuronal pretangles can be seen. Frequently present alongside AD pathology **Anatomic Distribution:** Characteristic progression starting from MTL structures and involving neocortex and brainstem late	Idiopathic
**Benign hereditary chorea type 2** ^a^	Adult‐onset nonprogressive chorea	4R‐tau	**Appearance:** NTFs, tufted astrocytes and argyrophilic threads. Unique neuronal cytoplasmic inclusion (non‐tau) in oculomotor nuclei **Anatomic Distribution:** Extensive tau pathology in the cortex, basal ganglia, brainstem, cerebellum	Genetic: linked to chromosome 8q21.3‐q23.3
**Beta‐propeller protein associated neurodegeneration (BPAN)**	Early‐onset seizures, developmental delay, behavioral problems (Rett syndrome–like phenotype) and ataxia. Later dystonia and parkinsonism. Pallidal iron accumulation on MRI	3R and 4R	**Appearance**: Tau threads, NFTs and pretangles **Anatomic Distribution**: Diffuse cortical, basal ganglia and brainstem involvement	Genetic: *WDR45* mutations
**Cerebrotendinous xanthomatosis** ^a^	Cataracts, atherosclerosis, neuropsychiatric symptoms, dementia, ataxia, seizures	4R‐tau	**Appearance:** 4R tau deposition with AGD‐like features **Anatomic Distribution:** Primarily limbic	Genetic: mitochondrial *CYP27A1* gene
**Chronic traumatic encephalopathy (CTE)**	Behavioral and mood changes, memory loss, cognitive impairment and dementia following repetitive head trauma	3R and 4R	**Appearance:** NFTs, threads and astrocytic inclusions; additional TDP‐43 and amyloid‐beta deposition found in a number of cases **Anatomic Distribution**: Clusters around small blood vessels of the cortex, typically at the sulcal depths; tau pathology with emphasis in the superficial cortex; CA4 and CA2 dendritic tau pathology	Environmental: repeated head trauma
**Corticobasal degeneration**	Variable clinical phenomenology broadly presenting either as CBS or as Richardson syndrome (RS). CBS presentations mostly take the form of asymmetric limb‐onset rigidity and apraxia, focal limb dystonia, myoclonus and cortical sensory deficits, whereas RS‐onset patients frequently manifest with gait disturbance, behavior change, vertical supranuclear gaze palsy, dysphagia, and symmetric parkinsonism	4R‐tau	**Appearance:** Tau pathology mainly comprises pretangles and thread‐like processes; Astrocytic plaques are typical **Anatomic Distribution:** In CBD‐CBS, tau pathology most severely involves primary motor cortex, putamen, globus pallidus externa, and subthalamic nucleus. In CBD‐RS, it primarily involves the brainstem structures, followed by the basal ganglia	Idiopathic
**Diffuse neurofibrillary tangles with calcification (Kosaka–Shibayama disease)**	Almost exclusively described in Japan. Initial AD‐like amnestic syndrome but often accompanied by behavior change (disinhibition, apathy), oral tendencies, parkinsonism, or gait disturbance. Neuroimaging: Fahr‐type calcifications	Mixed 3R and 4R	**Appearance:** Intracellular and extracellular AD‐like NFTs and glial fibrillary tangle **Anatomic Distribution:** Predominantly in limbic structures, which also commonly contain alpha‐synuclein, TDP‐43 pathology and plaque‐like structures	Idiopathic
**Down syndrome**	Trisomy 21 results in a poly‐symptomatic disorder, features of which include congenital cardiac defects, typical facial appearance, short stature, predisposition to leukemias and infections, mental retardation, and early‐onset AD	Mixed 3R and 4R	**Appearance:** Tau NFTs and amyloid plaques **Anatomic Distribution**: Similar to AD	Genetic: trisomy 21
**Cognitive decline in temporal lobe epilepsy**	Accelerated cognitive decline in temporal‐lobe epilepsy	Mixed 3R and 4R	**Appearance:** Hyperphosphorylated tau pathology in the form of neuropil threads, neurofibrillary tangles and pretangles; absence of amyloid‐beta plaques in most cases **Anatomic distribution:** Medial temporal lobe; can be AD‐like, CTE‐like, or an unusual subpial, axonal distribution	Idiopathic
**Familial British dementia**	A non‐Abeta cerebral amyloidosis demonstrating progressive cognitive impairment, spastic tetraparesis, and cerebellar ataxia	Mixed 3R and 4R	**Appearance:** Tau NFTs **Anatomic Distribution:** Tau mostly affecting the hippocampus. Deposition of amyloid protein (ABri) in blood vessels (amyloid angiopathy) and parenchyma, most prominently in the cerebellum and limbic structures	Genetic: Dominantly inherited mutations in the *BRI2* gene
**Familial Danish dementia**	A non‐Abeta cerebral amyloidosis characterized by early‐onset cataracts, followed later by deafness, ataxia, psychosis, and dementia	Mixed 3R and 4R	**Appearance:** Tau NFTs **Anatomic Distribution:** Tau mostly affecting the hippocampus. Deposition of amyloid protein (ABri) in blood vessels (amyloid angiopathy) and parenchyma, most prominently in the cerebellum and limbic structures	Genetic: Dominantly inherited mutations in the *BRI2* gene
**Focal cortical dysplasia type 2b**	Drug‐resistant childhood‐onset focal epilepsy	3R and 4R	Focal cortical dysplasia with dysmorphic neurons and balloon cells. Tau deposition confined to dysplastic neurons	Mutations in genes involved in mTOR pathway regulation found in some, but many remain idiopathic
**Frontotemporal lobar degeneration attributed to *MAPT* mutation**	Classic phenotypes include behavioral variant FTLD and primary progressive aphasia, although other features such as parkinsonism and motor neuron disease may dominate	3R, 4R, or mixed	**Appearance:** Variable tau pathology depending on mutation type; can resemble Pick's disease pathology with dominance of 3R tau, or CBD and PSP pathology with dominance of 4R tau **Anatomic Distribution:** Variable, depending on clinical syndrome	Genetic: *MAPT* mutations
**Frontotemporal lobar degeneration, Pick's disease**	Behavioral variant FTLD and less frequently primary progressive aphasia	3R	**Appearance:** 3R dominant neuronal and glial tau pathology **Anatomic Distribution:** Cortical (frontotemporal, medial temporal lobe)	Sporadic
**Ganglioglioma**	Epileptogenic malformation of cortical development frequently presenting with childhood seizures	–	NFTs within the dysmorphic neurons	Idiopathic
**Globular glial tauopathy**	Three clinical presentations recognized: 1. Frontotemporal dementia 2. Pyramidal weakness 3. Frontotemporal dementia/motor neuron disease overlap syndrome	4R‐tau	**Appearance:** Tau‐positive globular astrocytic inclusions (GAI) and globular oligodendrocytic inclusions (GOI). **Anatomic Distribution**: Varies according to clinical phenotype: 1. Frontotemporal regions 2. Motor cortex and corticospinal tracts 3. Frontotemporal regions, motor cortex, and corticospinal tracts	Idiopathic	
**Guadeloupean parkinsonism**	Axial‐predominant parkinsonism with early falls, frontal cognitive dysfunction and poor levodopa response in patients from Guadeloupe	4R	**Appearance:** Severe neuropil thread and NFT pathology. Severe nigral, pallidal, and thalamic neuronal loss with intense astrogliosis **Anatomic Distribution**: Cortex, basal ganglia, brainstem, and cerebellum	Unknown:? Genetic? Environmental
**Hemimegalencephaly**	Neonatal‐onset seizures. May be associated with neurocutaneous syndromes. Brain imaging showing hemimegalencephaly		Hyperphosphorylated tau deposition in dysplastic, hamartomatous cortical and hippocampal regions of the hemimegalencephalic hemisphere. Extensive lipid accumulation in neurons and astrocytes of the neocortex	Genetic: *AKT1* or *AKT3* gene mutations
**Heroin‐associated accelerated brain aging**	Cognitive dysfunction in heroin users	‐	**Appearance:** Tau neuropil threads or thin neurites. No Amyloid‐beta, Alpha‐synuclein, or TDP‐43 pathology **Anatomic Distribution:** Entorhinal cortex, locus coeruleus, amygdala as well as the frontal cortex and fusiform gyrus	Unknown
**Huntington's disease**	Clinical triad of dementia, hyperkinetic movements, and psychiatric symptoms. Tau deposition may underlie the development of cognitive impairment	Mixed 3R and 4R	**Appearance:** NFTs, nuclear rods, and tufted astrocytes. In addition, HTT aggregates, and senile plaques occasionally seen. **Anatomic Distribution:** Cortex, striatum, and in some cases, brainstem	Genetic: CAG trinucleotide repeat expansion in huntingtin gene
**Meningioangiomatosis**	Benign focal lesion of the leptomeninges and adjacent cerebral cortex characterized histologically by meningovascular proliferation and focal calcification. Most commonly presents with focal epilepsy		**Appearance**: NFTs **Anatomic Distribution**: Adjacent to focal brain lesions	May occur sporadically or in association with neurofibromatosis
**Motor neuron disease (rare)** ^a^	Most cases of familial and sporadic ALS are characterized by TDP‐43 deposition. However, a small proportion of sporadic (termed non‐Guamian MND with neurofibrillary tangles) and postencephalitic motor neuron disease is characterized by abnormal tau deposition	–	**Appearance:** NFTs **Anatomic Distribution:** Majority of pathology confined to medial temporal lobe structures, with lesser involvement of other cortical regions and brainstem nuclei (non‐Guamanian form) Diffuse involvement of spinal cord, brainstem nuclei, basal ganglia and sometimes cortex (postencephalitic form)	Idiopathic
**Myotonic dystrophy types 1 and 2**	Progressive muscle weakness, cataracts, endocrine dysfunction and cardiac conduction defects. Variable degrees of cognitive dysfunction	Unique class of tau isoforms lacking N‐terminal inserts	**Appearance:** NFTs **Anatomic Distribution:** Medial temporal lobe	Genetic: *DMPK* (DM1) and *ZNF9* (DM2) repeat expansions
**Niemann‐pick type C**	Ataxia, dementia, psychiatric manifestations, vertical supranuclear gaze palsy, gelastic cataplexy	Mixed 3R and 4R	**Appearance:** NFTs **Anatomic Distribution:** Diffuse tau deposition in MTL structures, neocortex, basal ganglia, and brainstem	Genetic: *NPC1* and *NPC2* gene mutations
**Nodding syndrome** ^a^	Neurodevelopmental regression, stereotyped head drops, seizures	Mixed 3R and 4R	**Appearance**: NFTs, pretangles and neuropil threads **Anatomic Distribution**: Cortex and brainstem with relative sparing of basal ganglia	Uncertain, possibly autoimmune reaction to *O. volvulus*
**Parkinson's disease, including genetic forms:** ***LRRK2*** ***WDR45*** ***PRKN***	Although a synucleinopathy, tau deposition is seen in Parkinson's disease. Clinically, both motor (tremor, bradykinesia, and rigidity) and nonmotor features will manifest at various stages of disease	–	**Appearance:** NFTs **Anatomic Distribution:** Substantia nigra	Idiopathic and genetic variants
**Postencephalitic parkinsonism**	Delayed, often asymmetric parkinsonism occurring either during the recovery phase after an encephalitic illness or many years after the original illness. Commonly associated with the outbreak of encephalitis lethargica, although the exact cause of PEP remains unknown	Mixed 3R and 4R	**Appearance:** Tau NFTs **Anatomic Distribution:** Neuronal loss, and astrocytosis in the SNR, and in the entorhinal cortex and hippocampus. Gross depigmentation of the SNR with relative preservation of the LC	Unknown:? Primary infection? Autoimmune/para‐infectious antibody‐mediated illness
**Primary age‐related tauopathy (PART)**	Spectrum ranging from normal to amnestic cognitive changes	3R and 4R	**Appearance:** NFT and/or neurites in the absence of amyloid plaque pathology **Anatomic Distribution**: predominantly in the medial temporal lobe, basal forebrain, brainstem, and olfactory areas (bulb and cortex)	Idiopathic
**Prion diseases** especially Gerstmann‐Sträussler‐Scheinker disease (GSS)	Rapidly progressive neurodegenerative disorders caused by misfolded prion proteins. Variable phenotypes, including rapidly progressive dementia, ataxia, parkinsonism, myoclonus, and sleep–wake disorders	Uncertain/variable	**Appearance:** Varied pathology including granular deposits, neuritic plaques, threads, NFTs and astroglial pathology.GSS usually shows severe intraneuronal tau pathology **Anatomic Distribution:** Again, varied, including ARTAG, PART, and various other distributions	Idiopathic, genetic, and acquired
**Progressive ataxia and palatal tremor (PAPT)** ^a^	Palatal tremor and cerebellar signs	Uncertain, either 4R or mixed 3R and 4R tau	**Appearance:** Neuropil threads and diffuse somatodendritic accumulation **Anatomic Distribution:** Most prominently in the inferior olivary nuclei (ION), and to lesser extents other brainstem nuclei and possibly the thalamus	Idiopathic
**Progressive supranuclear palsy (PSP)**	Axial rigidity, vertical supranuclear gaze palsy, cognitive decline	4R‐tau	**Appearance**: NFTs, pretangles, threads, oligodendroglial coiled bodies, and tufted astrocytes **Anatomic Distribution**: Cortex, basal ganglia, diencephalon, brainstem, and cerebellum	Idiopathic (some genetic predisposition, eg, H1 haplotype of *MAPT*)
**Senile dementia of the neurofibrillary tangle type (tangle‐only dementia)**	Late‐onset progressive dementia with predominent memory impairment	Mixed 3R and 4R	**Appearance:** Neurofibrillary tangles (predominantly extracellular‐ghost tangles) with striking absence of senile plaques **Anatomic Distribution**: NFTs mainly localized to the hippocampus, and to a lesser degree brainstem nuclei, but with sparing of cortex	Uncertain
**SLC9A6 mental retardation (Christianson syndrome)** ^a^	Males with neurodevelopmental delay, behavioral alterations, seizures, dystonia, and ataxia	Mixed 3R and 4R	Tau‐positive neuronal tangle‐like inclusions in the SNR, locus coeruleus, pontine nuclei, basal ganglia, thalami, cranial nerve nuclei, cerebral cortex, and hippocampus. Tau‐positive glial inclusions throughout the white matter. Astrocytic plaques seen in the cerebral white matter, thalamus, and brainstem	Genetic: X‐linked mutations in the *SCL9A6* gene
**Autosomal dominant spinocerebellar ataxia 11 and possibly 31** ^a^	Progressive ataxia with variable extracerebellar features (neuropathy, retinal degeneration, dystonia, cognitive decline, ophthalmoplegia)	Uncertain	**Appearance:** NFT, pretangles, and neuropil threads (SCA 11) **Anatomic Distribution:** Brainstem and basal ganglia (SCA 11)	Genetic: tau tubulin kinase‐2 (*TTBK2*) gene (SCA 11); *BEAN* gene (SCA 31)
**Subacute sclerosing panencephalitis**	Infrequent complication of measles virus infection characterized by behavioral and intellectual decline and periodic myoclonus following a latent postinfectious period	Uncertain	**Appearance**: Tau NFTs and (sometimes) straight filament (similar to PSP). In addition, severe neuronal loss, gliosis, demyelination, perivascular inflammation, Cowdry type‐A and type‐B bodies **Anatomic distribution:** Present in cortex and hippocampus, and to a lesser degree in the brainstem. Tau deposition appears to correlate with disease duration, being generally present if duration is >1 year	Postinfectious
**Early‐onset refractory seizures and progressive neurological decline attributed to *SYNJ1* mutation** ^a^	Recessive inheritance of *SYNJ1* variants are reported in 2 clinical syndromes: 1. Early‐onset intractable epilepsy with cognitive decline 2. Early‐onset Parkinsonism with seizures and dystonia Tau pathology has been reported in 1	‐	**Appearance:** Tau NFTs **Anatomic Distribution:** Tau deposition predominantly in nigral neurons	Genetic: *SYNJ1* mutation
**Traumatic basal ganglia injury with secondary dystonia** ^a^	Single case: Posttraumatic focal dystonia resulting from head injury related basal ganglia lesion	Uncertain	**Appearance:** NFTs, neurites, and tau astroglial pathology **Anatomic Distribution:** Basal ganglia	Posttraumatic
**Tuberous sclerosis complex**	Multisystem neurocutaneous syndrome characterized by hamartoma formation in various organs, neuronal migration defects, and tumors. Presentations are highly variable but neurologic presentations generally comprise seizures and/or intellectual disability	Uncertain	Tau deposition seen to varying degrees in cortical tubers	Genetic: Autosomal dominant mutations in *TSC1* and *TSC2* genes
**X‐linked parkinsonism with spasticity** ^a^	Young‐onset parkinsonism with spasticity	4R‐tau	**Appearance:** NFTs and neurites **Anatomic Distribution**: Medial temporal lobe and SNR Diffuse Abeta deposits in the neocortex and limbic system, with neuritic plaques confined to the medial temporal lobe; no Lewy bodies. Plaque‐like structures in the striatum	Genetic: X‐linked mutations in the *ATP6AP2* gene

^a^ Tau pathology so far only demonstrated in small numbers of case reports and/or unclear, potentially coincidental association with tau pathology.

NFT, neurofibrillary tangles; ADCY5, adenylate cyclase 5; CTE, chronic traumatic encephalopathy; WM, white matter; GM, gray matter; OSA, obstructive sleep apnea; MCI, mild cognitive impairment; CBD, corticobasal degeneration; MTL, medial temporal lobe; SNR, substantia nigra; LC, locus coeruleus; MRI, magnetic resonance imaging; TDP‐43, TAR DNA binding protein‐43; CBS, corticobasal syndrome; mTOR, mammalian target of rapamycin; FTLD, frontotemporal lobar degeneration; HTT, huntingtin; MND, motor neuron disease; PEP, postencephalitic parkinsonism; SCA, spinocerebellar ataxia.

Indeed, as the number of disparate disorders bearing tau pathology increases, the less likely it becomes that tau is the primary instigator or mediator of disease in all. Moreover, the recognition of a number of aging‐related tauopathies (aging‐related tau astrogliopathy; primary age related tauopathy), sometimes in patients without clinical deficits, further supports the theory that on occasion, tau deposition may be a late or secondary phenomenon or may merely reflect processes associated with normal aging and life insults.^1^, ^2^

## Brief Overview of Tau

Tau is a critical microtubule‐associated protein subserving varied functions ranging from maintenance of cytoskeletal architecture and axonal transport to neurite polarization and DNA protection.^3^ A total of 6 tau isoforms exist, generated through alternate splicing of exons 2, 3, and 10 of the *MAPT* gene on chromosome 17; inclusion or exclusion of exon 10 produces tau with either 3 (3R) or 4 (4R) microtubule‐binding domains, the normal 1:1 ratio of which can become skewed in various disease states.^3^ In disease, tau becomes misfolded and physiochemically abnormal and accumulates as insoluble inclusions in neurons and/or glia, the neuropathologic detection of which denotes the condition as a “tauopathy.” Recent evidence has uncovered “prion‐like” cell‐to‐cell tau propagation along the neuro‐anatomic connectome and strain‐specific tau pathologic inclusions and seeding characteristics.^3^, ^4^, ^5^

In addition to the division into primary (where tau is considered the sole and/or primary mediator of neurodegeneration) and secondary (where tau coexists with other pathology) forms, disease‐specific signatures for each tauopathy exist based on which cell types bear inclusions (neurons, glia, or both), their morphological appearances and anatomic distribution, and the ratio of 3R:4R tau.^3^, ^6^

Herein we discuss recent movement disorder additions to the field of tauopathies. Deciding which disorders to include was difficult, as some subjectivity exists regarding what constitutes a “new” or “unexpected” finding. We tried to focus on disorders that would be of interest to practicing clinicians and group these according to likely disease pathomechanisms.

## Hereditary Disorders

### Beta‐Propeller Protein Associated Neurodegeneration (BPAN)

Described in 2012, this neurodegenerative disorder with brain iron accumulation mostly results from de novo mutations in the *WDR45* gene.^7^ Despite being x‐linked, BPAN is uncommon in males, likely because of the nonviability of affected conceptuses.^7^ Clinical characteristics include early‐onset seizures, global developmental delay, ataxia, and behavioral problems often with a Rett syndrome–like phenotype.^7^ In adolescence, seizures become less prominent, parkinsonism and dystonia appear, and dementia progresses.^7^

Neuropathology shows widespread Alzheimer‐like tau pathology throughout the cortex, basal ganglia, brainstem, and cerebellum.^8^ The notable absence of alpha‐synuclein pathology helps to differentiate BPAN from other neurodegenerative disorders with brain iron accumulation, particularly phospholipase A2 group VI‐associated neurodegeneration and mitochondrial membrane protein associated neurodegeneration, while tau anatomic distribution is quite distinct from that seen in Alzheimer's disease (AD).^7^

### Adenylate Cyclase 5 (ADCY5)–Related Dyskinesia

ADCY5‐related dyskinesia is a childhood‐onset choreodystonic movement disorder caused by mutations in the ADCY5 family of striatal‐specific enzymes, which convert ATP to the second messenger cyclic adenosine monophosphate.^9^ Associated symptoms may include axial hypotonia, limb hypertonia, cognitive impairment, depression, attention‐deficit hyperactivity disorder, and psychosis. Oculomotor apraxia and nocturnal ballistic bouts may be seen.^10^

Neuropathologic features of the disorder have yet to be clearly defined. Recently, the first neuropathological examination of a patient with a genetically confirmed (p.M1029K) *ADCY5* gene mutation who died at the age of 46 years was reported.^11^ This showed widespread (mixed 3R/4R) tau pathology, including neurofibrillary tangles, neuritic tau, and glial tau deposition involving the cerebral cortex, midbrain, thalamus, and hippocampus. Sulcal perivascular astroglial tau inclusions were identified, raising questions about whether this could have represented chronic traumatic encephalopathy pathology from severe choreodystonic head movements, but the pathology was not typical for this disorder.^11^

### Benign Hereditary Chorea (BHC) Type 2

Most BHC syndromes result from mutations in either the thyroid transcription factor 1 (*TITF1*)^12^or *ADCY5* genes.^13^ BHC type 2, the rarest benign chorea syndrome, was described in 2007 in 2 Japanese families with adult‐onset dominantly inherited progressive chorea and mapped to chromosome 8q 21.3‐q23.3.^14^ Neuropathological findings in a single case of BHC type 2 included presence of neurofibrillary tangles and threads and 4R immunoreactive tufted astrocytes (a hallmark feature of progressive supranuclear palsy) affecting the cortex, basal ganglia, brainstem, and cerebellum.^15^ Of note, the reported patient was 83 years old at the time of death, hence it remains to be seen if tau pathology in BHC is a specific feature or rather a concomitant age‐related pathology or unrelated primary tauopathy.

### Autosomal Dominant Spinocerebellar Ataxias (SCAs)

SCAs are dominantly inherited degenerative disorders characterized principally by progressive ataxia, often accompanied by extracerebellar symptoms including parkinsonism, pyramidal signs, retinal degeneration, and peripheral neuropathy.^16^

Tau deposition has been reported in both SCA 11 (1 patient, age at death uncertain but >77 years) and SCA 31 (1 patient, aged 76 years).^16^, ^17^, ^18^ In the former, neurofibrillary tangles, neuropil threads, and tau‐positive neurites involved various brainstem regions and the basal ganglia.^17^ In the latter, tau may simply represent coincidental AD pathology.^18^

### Huntington's Disease (HD)

HD is a dominantly inherited neurodegenerative disorder classically producing the triad of cognitive decline, psychiatric symptoms, and hyperkinetic movements.^19^ Expansion of the CAG trinucleotide repeat in the huntingtin (*HTT*) gene elongates the protein's polyglutamine tract, causing misfolding, aggregation, and impairment of various cellular processes.^19^

Along with other misfolded proteins, such as alpha‐synuclein and TAR DNA binding protein‐43, tau deposition is increasingly recognized as a feature of HD neuropathology, taking the form of neurofibrillary tangles predominantly in medial temporal lobes as well as nuclear tau rods (Lucas rods).^20^, ^21^ Tau pathology, which may be responsible for the development of cognitive symptoms in HD,^21^ increases with increasing disease stage, suggesting a direct role for mutant *HTT* in formation of insoluble tau.^20^

## Autoimmune Disorders

### Anti‐IgLON5 Disease

Anti‐IgLON5 disease is a nonparaneoplastic antibody‐associated disorder classically presenting in the sixth to seventh decade of life with disordered sleep (parasomnias, obstructive sleep apnea, stridor, hypoventilation), cognitive decline, bulbar symptoms, and ataxia. Parkinsonism, sometimes assuming a progressive supranuclear palsy–like phenotype, can be present, as can chorea.^22^ The rate of progression is slow, at times decades from diagnosis until death. Response to immunotherapy may be seen.^23^

Neuropathologic findings in the disorder were considered relatively homogeneous, showing almost exclusively neuronal 3R‐tau and 4R‐tau deposition primarily in the brainstem tegmentum and hypothalamus, following a cranio‐caudal gradient to the level of the cervical spinal cord.^22^ However, the recent report of a case of symptomatic anti‐IGLON5 disease without brainstem tau pathology questions the pathogenic role of tau in this disorder.^24^

## Idiopathic/Environmentally Mediated Disorders

### Progressive Ataxia and Palatal Tremor (PAPT)

The PAPT syndrome is characterized by palatal tremor along with progressive cerebellar signs^25^; cerebellar atrophy and hypertrophic olivary degeneration are frequent neuroimaging findings. PAPT is classified as either “sporadic” or “familial.”^25^ Familial PAPT is usually a consequence of adult‐onset Alexander's disease, although rarer causes include autosomal dominant SCA type 20 and DNA polymerase gamma (*POLG*) mutations.^25^ “Sporadic” PAPT subsumes the nonfamilial conditions, including celiac disease and cerebrotendinous xanthomatosis, although most cases remain idiopathic.

Two separate reports (on 3 patients, aged 75, 80, and 89 years at the time of autopsy) have identified tau deposits in idiopathic PAPT. Its distribution differed, but generally comprised somatodendritic inclusions in the inferior olives, nigra, locus coeruleus, red nucleus, and thalami alongside cortical and/or hippocampal tau pathology that was interpreted as an incidental finding related to aging.^26^, ^27^

### Globular Glial Tauopathies (GGT)

The GGT are rare 4R‐tauopathies characterized neuropathologically by widespread globular glial inclusions.^28^ Various neuropathologic subtypes of GGT have been defined:

- Type I GGT classically presents with fronto‐temporal dementia, reflecting predominant fronto‐temporal distribution of pathology.
- Type II GGT generally demonstrate prominent pyramidal involvement, reflecting motor cortex pathology with corticospinal tract degeneration.
- Type III GGT is essentially a combination of types I and II, exhibiting combined involvement of the motor cortex and frontotemporal regions and consequent symptomatic overlap.

GGT types II and III may demonstrate extrapyramidal features and can be misdiagnosed as either progressive supranuclear palsy or corticobasal syndrome, sometimes with “typical” neuroimaging.^29^, ^30^ Clinical clues in these cases include prominent upper motor neuron signs, with most cases essentially representing primary lateral sclerosis overlap syndromes.^29^, ^30^ Tau strains in GGT demonstrate unique, potent seeding characteristics that appear to be a primary driver of the neurodegenerative process.^31^

### Traumatic Brain Injury Producing Secondary Dystonia

A single case report of a 78‐year‐old male patient demonstrated tau pathology potentially underlying the development of secondary dystonia following focal traumatic brain injury. Neuropathology, in addition to chronic traumatic encephalopathy–like tau deposition in the frontal cortex (the likely site of impact), and histologic evidence of PART, showed evidence of focal right basal ganglia neuronal and glial tau deposition, likely accounting for left hand dystonia.^32^ The report in question should, however, be interpreted with caution, especially given the self‐confessed clinical uncertainties about the diagnosis of dystonia.^32^

### Nodding Syndrome (NS)

NS is an enigmatic epidemic neurologic disorder affecting children in East Africa.^33^ Joining other geographical clusters of tauopathy such as Guadeloupean parkinsonism and parkinsonism–dementia complex of Guam, the disorder manifests between 3 and 18 years of age with stereotyped head drops, seizures, neurocognitive regression, and slow, inexorable progression until death.^33^ Its etiology is uncertain. Theories include intentional poisoning of water sources, exposure to chemicals, neurotropic viruses, and parainfectious autoimmune disorders.^34^

Recent neuropathologic examination of 5 patients who had died of NS showed neuronal tau deposition (pretangles and neuropil threads) predominantly in the cortical and brainstem regions, with relative sparing of basal ganglia.^35^ However, epilepsy is one of the clinical features of NS, and given that tau pathology is well known to occur in long‐standing epileptics,^36^ it could be that the observed changes are secondary to poorly controlled seizures.

## Discussion

Fundamentally, all tauopathies are characterized by deposition of physio‐chemically abnormal, misfolded tau within neurons and/or glia. The upstream pathophysiologic abnormalities leading to such conformational changes are vast. They include alterations in amino acid sequences within the *MAPT* gene, altered transcription, multiple posttranslational influences (but especially hyperphosphorylation), head trauma, ischemia, and oxidative stress. Therefore, although classifying disorders together according to their neuropathologic similarities is informative, it can convey the wrong message that these diseases all function in similar ways and that disease modification can be applied using a “one size fits all” approach. This is incorrect, and perhaps may be one reason for the disappointing results obtained thus far from disease‐modification treatment trials in tauopathies.

With new diseases come new insights about disease mechanisms. Nowhere is this more true than with the new and unexpected tauopathies. Although many of the disorders described previously reflect experience from small numbers of cases and therefore need to be replicated, studying these offers great potential to further our understanding of disease physiology and disclose novel disease‐modifying treatment targets.

The aforementioned disorders may critically affect tau structure at various levels. For example, *ADCY5* mutations influence cyclic adenosine monophosphate production; in turn, cyclic adenosine monophosphate–dependent protein kinases regulate (among other things) tau phosphorylation and exon 10 splicing.^37^ In BPAN, defective autophagy likely impairs clearance of proteins, including tau.^38^ Moreover, iron dyshomeostasis in this disorder may influence tau phosphorylation and aggregation.^39^ Posttraumatic dystonia, similar to chronic traumatic encephalopathy, reflects the fact that head trauma and shear forces can influence tau folding.^40^ The SCA 11 gene product *TTBK2* encodes a kinase that putatively phosphorylates tau and tubulin proteins. SCA 31 affects the *BEAN* gene, coding for a protein that marks defective tau for subsequent degradation. In HD, mutant *HTT* protein can promote tau hyperphosphorylation and disrupt normal tau splicing, shifting the balance in favor of 4R‐tau isoforms.^21^ Anti‐IGLON5 disease, and possibly NS, offer an opportunity to study the interplay of autoimmunity with later neurodegeneration.

Furthermore, these cases illustrate the importance of untangling what truly represents a tauopathy. Should scant amounts of tau deposition be enough to merit classification of a disorder as a tauopathy and to ascribe disease initiation and/or progression to the observed neuropathologic inclusions? How well defined and reproduced should the neuropathology be to merit this definition? Should there always be a corresponding clinical syndrome? Could tau in some cases be an innocent bystander or a reflection of normal aging?

Further systematic clinical, neuropathologic, and molecular characterization of tauopathies is needed to address these questions and hopefully concomitantly allow the development of targeted disease‐modifying therapies.

## Author Roles

1) Research project: A. Conception, B. Organization, C. Execution; 2) Statistical Analysis: A. Design, B. Execution, C. Review and Critique; 3) Manuscript: A. Writing of the first draft, B. Review and Critique.

E.M.: 1A, 1B, 1C, 3A, 3B

B.B.: 1C, 3A, 3B

Z.J.: 1C, 3A, 3B

R.E.: 1C, 3A, 3B

A.L.: 1C, 3A, 3B

K.P.B.: 1C, 3A, 3B

## Disclosures

**Ethical Compliance Statement:** The authors confirm that the approval of an institutional review board/ patient consent was not required for this work. We confirm that we have read the Journal's position on issues involved in ethical publication and affirm that this work is consistent with those guidelines.

**Funding Sources and Conflicts of Interest:** None of the authors report any conflict of interest or financial disclosures in relation to this article.

**Financial Disclosure for previous 12 months:** B.B. was supported by the Robert Bosch Foundation. R.E. received honoraria for speaking at meetings from UCB and the International Parkinson's Disease and Movement Disorder Society. He receives royalties from the publication of *Case Studies in Movement Disorders—Common and Uncommon Presentations* (Cambridge University Press, 2017). Z.J. is supported by the Department of Health's National Institute for Health Research Biomedical Research Centre's funding scheme. K.P.B. holds research grants from EU Horizon 2020 and has received honoraria to speak at meetings or to attend advisory boards from Ipsen, Cavion, Allergan, Teva Lundbeck, and Bial pharmaceutical companies. He also receives royalties from Oxford University Press and a stipend for *Movement Disorders Clinical Practice* editorship.
